# Plasma concentrations of Gas6 (growth arrest specific protein 6) and its soluble tyrosine kinase receptor sAxl in sepsis and systemic inflammatory response syndromes

**DOI:** 10.1186/cc9233

**Published:** 2010-08-23

**Authors:** Carl Ekman, Adam Linder, Per Åkesson, Björn Dahlbäck

**Affiliations:** 1 Department of Laboratory Medicine, Division of Clinical Chemistry, Lund University, Skåne University Hospital, Entrance 46, SE-20502 Malmö, Sweden; 2 Department of Clinical Sciences, Division of Infection Medicine, Lund University, Skåne University Hospital, Tornav 10, SE-221 84 Lund, Sweden

## Abstract

**Introduction:**

Gas6, the protein product of the growth arrest specific gene 6, is present in human circulation at subnanomolar concentrations. It is secreted by endothelial cells and is important for the activation of endothelium during inflammation. Axl, the tyrosine kinase receptor for Gas6, is also present in endothelium and can be cleaved and released into the circulation. The soluble of form Axl (sAxl), which is present in plasma, can bind Gas6 and inhibit Axl-mediated cell signalling.

**Methods:**

We have developed reproducible and accurate enzyme-linked immunosorbent assays for both Gas6 and sAxl and used them to investigate plasma samples from 70 patients with severe sepsis, 99 patients with sepsis, 42 patients with various infections causing fever but no systemic inflammatory response syndrome (SIRS), 20 patients with SIRS without verified infection, and 100 blood donors that served as controls. Correlations between Gas6 and sAxl concentrations and other commonly used analytes were investigated.

**Results:**

The patients with severe sepsis, sepsis, infection or SIRS had all increased concentrations of Gas6, approximately double compared to what was found in the controls. The concentrations of sAxl were also increased in the patient groups compared to the controls. Gas6 correlated with C-reactive protein, procalcitonin and interleukin 6, whereas sAxl correlated to bilirubin and procalcitonin.

**Conclusions:**

We can confirm results of earlier studies showing that circulating Gas6 is increased in sepsis and related syndromes. sAxl is increased, but less pronounced than Gas6. The concentrations of Gas6 and sAxl correlate with a number of inflammatory markers, suggesting a role in systemic inflammation.

## Introduction

Gas6 is a vitamin K-dependent protein, which was initially described as a protein expressed during growth arrest [1]. It is structurally related to the anticoagulant protein S, the two proteins having 44% amino acid identity [2]. Both Gas6 and protein S bind the TAM family of tyrosine kinase receptors that comprises Tyro3, Axl and Mer [3]. The binding of Gas6 to Axl induces Axl phosphorylation and activation of the PI3 kinase/Akt pathway, which has prosurvival and antiapoptotic effects [4]. Gas6 has also been shown to be important for phagocytosis of apoptotic cells [5,6]. Gas6 can regulate the inflammatory response by downregulating TNFα, IL-6 and interferon secretion in dendritic cells [7], and, interestingly, animals lacking the TAM family of receptors develop autoimmune diseases [8]. Gas6 and Axl are involved in activating the endothelium in response to inflammation, increasing the leucocyte extravasation and rejection of transplants [9].

The membrane-bound Axl can be shed from the cell membrane as a result of proteolysis, and Axl is therefore present in circulation in a soluble form (sAxl) that consists of the extracellular region of the protein. The presence of sAxl in plasma has been demonstrated in mice [10], and we have recently found that sAxl is normally in excess of Gas6 and that Gas6 is bound to sAxl in normal human serum and plasma [11].

Sepsis includes a complex clinical syndrome, the systemic inflammatory response syndrome (SIRS), resulting from a harmful or damaging host response to infection. The incidence of sepsis is approximately 3 cases per 1,000 individuals and the overall mortality is 10 to 50% [12]. SIRS can also develop independently of any infection, for example, in cases of pancreatitis, trauma, or immune complex disease [13]. Two previous studies have found increased concentrations of Gas6 in sepsis, but its relation to sAxl concentrations has not been investigated [14,15]. The aim of this study was to investigate the Gas6 and sAxl concentrations in plasma in a large cohort of patients with sepsis and related inflammatory syndromes. We can report that the plasma Gas6 was increased approximately two-fold in all patient groups.

## Material and methods

### Gas6 and sAxl ELISAs

Blood samples for the analyses of plasma proteins and lactate were collected from subjects at enrollment in the study in 5 ml plastic vacutainer tubes containing 0.5 ml 0.129 mol/l sodium citrate, as previously described [16]. The Gas6 [17] and sAxl [11] ELISAs have been described earlier. In short, maxisorb (Nunc) plates were coated with a catching polyclonal antibody before blocking with 3% fish gelatin in 50 mM Tris-HCL, 150 mM NaCl, pH 7.4 with 0.1% Tween 20. The samples were diluted in the blocking buffer and incubated overnight before washing and detection with a biotinylated secondary antibody. The signal was amplified using ABC/HRP (Dako, Glostrup, Denmark) and visualized with 1,2-phenylenediamine dihydrochloride and hydrogen peroxide. Sulphuric acid was added to stop the reaction before measuring the absorbance at 490 nm. The absorbance of the samples was compared to a standard curve prepared by a dilution series with known amounts of the respective protein.

### Study population

The study cohort has previously been described in detail [16]. Briefly, 232 patients were enrolled in a prospective study at the Clinic for Infectious Diseases, University Hospital, Lund, Sweden. The inclusion criteria were fever (≥38°C) and a suspected infection. Only adults (≥18 years of age) were included. The blood sampling was performed within 12 hours after admission to the hospital. The ethics committee of Lund University approved the project protocol, and informed consent was obtained from all patients or their close relatives.

Based on the presence of SIRS criteria (body temperature ≥38°C, WBC >12 × 10^9^/l or <4 × 10^9^/l, pulse rate >90/minute and respiratory rate >20/minute [13]), or a significant hypotension (a systolic blood pressure of <90 mmHg or a fall of >40 mmHg from baseline), presence or absence of organ failure, and the final diagnosis, the patients were categorized into various groups. The criteria were those proposed by the American College of Chest Physicians/Society of Critical Care Medicine [13].

The patients were divided into the following groups: Severe Sepsis, Sepsis, Infection and SIRS. Severe sepsis was defined as an infectious disease, at least two SIRS criteria, and the presence or development of hypotension and/or organ failure within 24 h of the collection of the blood samples. Sepsis was defined as an infectious disease, at least two SIRS criteria, but no presence or development of organ failure. Infection was defined as an infectious disease without SIRS. SIRS was defined as a non-infectious disease with at least two SIRS criteria. Renal failure was defined using the RIFLE criteria [18].

### Statistical analysis

Nonparametric tests were used throughout the study. The Mann-Whitney U test was used for evaluating the difference between different groups, and Spearman's rank correlation coefficient for evaluating correlations. For all tests *P *< 0.05 was considered significant. Graphpad Prism 4.0 (Graphpad software, La Jolla, CA, USA) was used for statistics.

## Results

### Patients

Two hundred and thirty-two patients were included. Seventy patients were diagnosed with severe sepsis, 99 patients with sepsis, 43 patients with infection without SIRS, and 20 patients with SIRS without infection. Detailed patient demographic data and diagnoses have been presented elsewhere [16]. Pneumonia and urinary tract infections were common and also overrepresented in the severe sepsis and the sepsis groups. Infected patients without SIRS suffered mostly from upper respiratory infections. The 20 patients with non-infectious SIRS suffered from various diseases such as vasculitis, cardiac failure, gastrointestinal bleeding, pulmonary embolism and pancreatitis. The over-all mortality rate was 3.4%. In the severe sepsis group, the mortality rate was 10%, and out of the 26 patients with septic shock, 19% died.

### Plasma levels of Gas6 and sAxl

**Figure 1:**
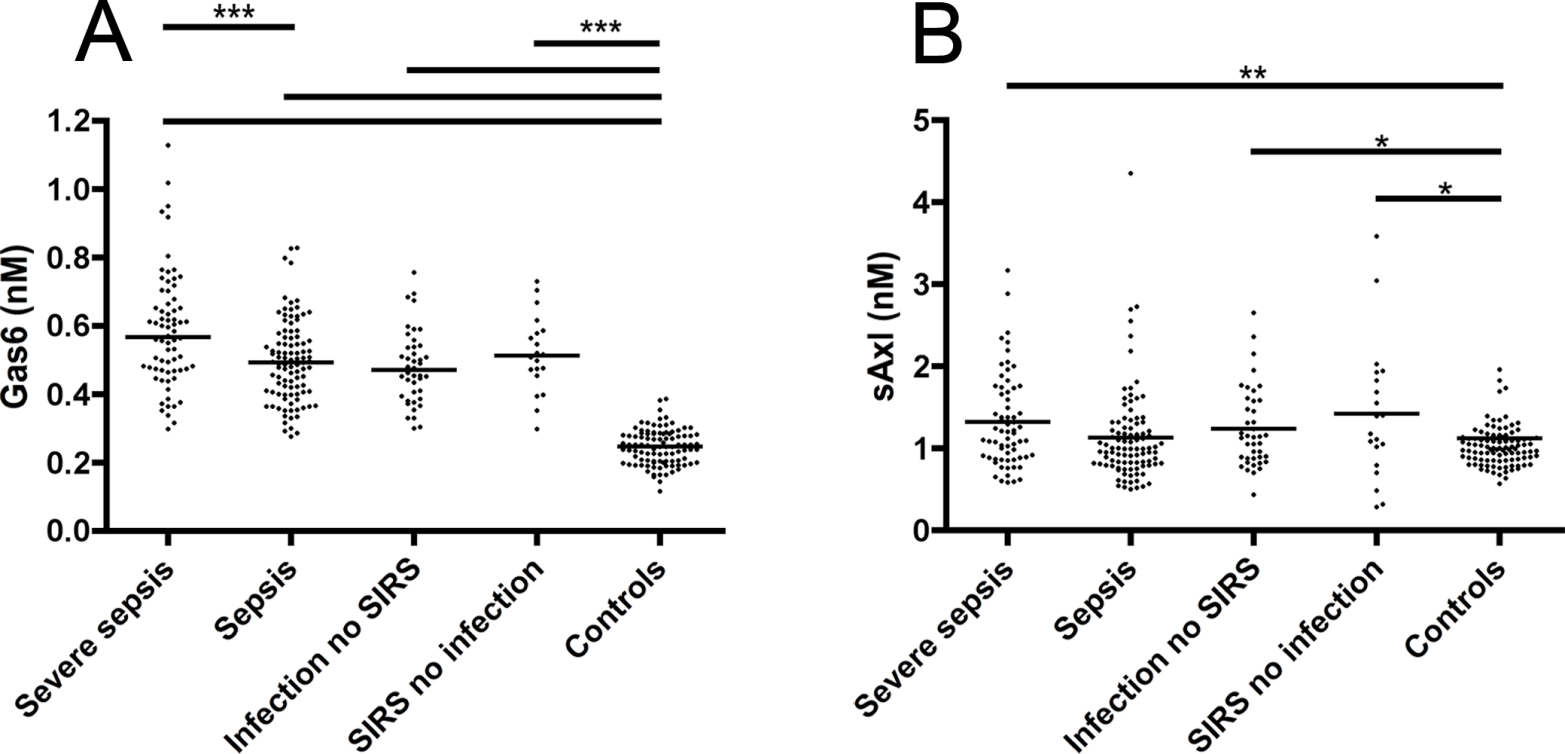
**Comparison of Gas6 and sAxl plasma concentrations in the different patient groups**. **(a) **The Gas6 concentrations in patient and control samples; **(b) **the sAxl concentrations. The statistical significances of the differences between the groups were evaluated with the Mann-Whitney test. *= *P *< 0.05, ** = *P *< 0.01, *** = *P *< 0.001.

The plasma concentrations of Gas6 and sAxl were determined in the acutely ill patients who were found to suffer from severe sepsis, sepsis, infections without SIRS, or SIRS without infection (Figure 1). When compared to the controls, all patient groups had significantly increased plasma concentrations of Gas6, the median Gas6 concentration being 0.58, 0.50, 0.48 and 0.52 nM for the patient groups and 0.25 nM for the controls. The patients with severe sepsis had significantly increased Gas6 concentrations when compared to the sepsis group (Figure 1a). The median plasma sAxl concentrations were 1.19, 1.00, 1.14, 1.29 and 0.99 nM, respectively. There were also statistical differences in sAxl between the controls and the patient groups, but they were not close to the significance levels observed for Gas6. There were several individuals with very high sAxl concentrations in the patient groups, but the differences between the groups were less pronounced than what was found for Gas6 (Figure 1b).

Both Gas6 and sAxl concentrations correlated with previously measured analytes [16]. When combining all the 232 samples in one group, the concentration of Gas6 correlated with those of IL-6, procalcitonin and number of failing organs. Also sAxl concentrations correlated to Gas6, procalcitonin and number of failing organs (Table 1).

**Table 1:** Correlations observed between Gas6 and sAxl in all patient groups combined

Correlations in all patient groups combined		
Gas6 correlations	r	*P*-value
Interleukin 6	0.36	< 0.0001
Procalcitonin	0.34	< 0.0001
Number of failing organs	0.27	< 0.0001
Bilirubin	0.26	0.0092
INR	0.26	0.0113
Thrombocytes	-0.24	0.0021
Breathing frequency	0.18	0.0052
C-reactive protein	0.18	0.0054
Blood pressure	-0.17	0.0084

**sAxl correlations**	**r**	***P*-value**

Gas6	0.26	< 0.0001
Procalcitonin	0.17	0.0108
Number of failing organs	0.16	0.0178
Leucocytes	-0.15	0.0262
C-reactive protein	-0.14	0.0404

The strength of correlation was evaluated with Spearman's rank correlation test. INR, international normalized ratio.

When evaluating the severe sepsis and sepsis groups separately, Gas6 correlated to IL-6, bilirubin, INR, procalcitonin and number of failing organs. sAxl correlated to bilirubin, Gas6, number of organs in failure and inversely to C-reactive protein (Table 2).

**Table 2:** Correlations between Gas6 or sAxl and other analytes in the combined severe sepsis and sepsis group

Correlations for severe sepsis and sepsis		
Gas6 correlations	r	*P*-value
Interleukin-6	0.36	< 0.0001
Bilirubin	0.31	0.0035
INR	0.31	0.0037
Procalcitonin	0.30	< 0.0001
Number of failing organs	0.28	0.0003
Thrombocytes	-0.26	0.0025
Lactate	0.21	0.0062
Blood pressure	-0.17	0.0307
Breathing frequency	0.15	0.0477

**sAxl correlations**	**r**	***P*-value**

Bilirubin	0.27	0.0149
Gas6	0.26	0.0007
Number of failing organs	0.21	0.0073
C-reactive protein	-0.20	0.0119
Lactate	0.18	0.0255
Procalcitonin	0.16	0.0356

The strength of the correlations were evaluated with the Spearman's rank correlation test. INR, international normalized ratio.

Gas6 was higher in patients with organ failure, kidney failure and in patients receiving intensive care, whereas sAxl was higher in patients with organ failure. There was a non-significant trend towards higher Gas6 in patients that did not survive (Table 3).

**Table 3:** Median Gas6 and sAxl concentrations in patients grouped depending on organ failure, intensive care or death

Condition	n pos	Gas6 pos	Gas6 neg	*P*-value
Organ failure	81	0.56	0.49	**< 0.0001**
Kidney failure	33	0.56	0.50	**0.0021**
Intensive care	17	0.58	0.50	**0.0380**
Death	10	0.56	0.50	0.1414

**Condition**	**n pos**	**sAxl pos**	**sAxl neg**	***P*-value**

Organ failure	77	1.18	1.05	**0.0216**
Kidney failure	30	1.20	1.06	0.2169
Intensive care	15	1.12	1.07	0.3664
Death	9	1.12	1.08	0.4883

n positive indicates how many of the 232 patients who were included in the positive group. Bold indicates that the distribution was statistically significant using the Mann-Whitney U test. Gas6 and sAxl concentrations are in nM.

## Discussion

In this study, we have determined the Gas6 and sAxl concentrations in a large number of patients with sepsis and related inflammatory conditions. Our data support the previous reports of increased Gas6 during sepsis [14,15], and we find correlations between Gas6 or sAxl concentrations and degree of organ damage. These results suggest that the production of Gas6 is strongly up-regulated during severe inflammatory reactions but also during milder infections.

Gas6 is bound to sAxl in plasma and there is a molar surplus of sAxl compared to Gas6 [11]. The binding of sAxl to Gas6 presumably inhibits the ability of Gas6 to bind and stimulate cell surface bound Axl suggesting that sAxl in blood inhibits circulating Gas6. The relative increase in Gas6 concentration in the patients is higher than that of sAxl, suggesting that increased Gas6-mediated cellular signaling occurs during sepsis, but it remains to be determined in which cells the increased signaling occurs.

The circulating Gas6 is presumably derived locally in the affected tissue and the doubling in plasma we observe during inflammatory reactions suggests that the Gas6 synthesis may be highly increased in the locus of inflammation. The source of the circulating Gas6 is not clear, but both endothelium and different leukocytes have been found to release Gas6 [9,14].

The sAxl concentration is changed in the patients compared to the controls, but not to the same magnitude as Gas6. As Axl is ubiquitously expressed, several cell- and tissue-types may be the source of the sAxl in sepsis. Both Axl and Mer have been shown to be shed under the influence of PMA and LPS [10,19].

Gas6 correlates with IL-6, procalcitonin and the number of organs failing, whereas sAxl correlates to bilirubin, Gas6, procalcitonin and number of failing organs, indicating that both Gas6 and sAxl are increased in inflammatory states.

Gas6 and sAxl are increased during organ failure, and Gas6 is increased in patients receiving intensive care or experiencing kidney failure, again indicating that Gas6 is increased in severe inflammatory states. There is a non-significant trend to higher Gas6 in patients not surviving, but the low number of non-surviving patients makes the analysis uncertain.

Due to the large increase of Gas6, Gas6 induced signaling is presumably increased during sepsis and related inflammatory conditions. Gas6 is involved in several systems, which are active during sepsis. This includes phagocytosis [6], maturation of immune cells [20], endothelial activation [9] and immunoregulation [7].

The main effects of the Gas6 signaling in sepsis remain to be determined.

## Conclusions

We have measured the Gas6 and sAxl plasma concentrations in a large cohort of patients with severe sepsis, sepsis, milder infections, and SIRS without infection, and found that Gas6 increases in all patient groups, and the concentration correlates with disease severity and organ dysfunction. sAxl is also increased, but it does not follow the two-fold increase observed for Gas6, indicating increased Gas6 signalling during sepsis and related inflammatory conditions.

## Key messages

• Gas6 plasma concentrations are increased in patients with sepsis, SIRS and infections compared to controls.

• Gas6 behaves as an acute phase protein.

## Abbreviations

ELISA: enzyme linked immunosorbent assay; Gas6: growth arrest specific 6; IL-6: interleukin-6; LPS: lipopolysaccharide; PMA: phorbol 12-myristate 13-acetate; sAxl: soluble Axl; SIRS: systemic immune response syndrome; TAM: Tyro3, Axl, Mer; TNFα: tumor necrosis factor alpha.

## Competing interests

The authors declare that they have no competing interests.

## Authors' contributions

CE performed the ELISAs, assisted in analysis of the data and wrote parts of the manuscript. AL participated in the design of the clinical study, included and followed patients, assisted in analysis of the data and wrote parts of the manuscript. PÅ participated in the design of the clinical study, included and followed patients and wrote parts of the manuscript. BD initiated the study, participated in the data analysis and wrote parts of the manuscript. All authors approved the final version of the manuscript.

## References

[B1] Schneider C King RM Philipson L Genes specifically expressed at growth arrest of mammalian cells Cell 1988 54 787 793 10.1016/S0092-8674(88)91065-3 3409319

[B2] Manfioletti G Brancolini C Avanzi G Schneider C The protein encoded by a growth arrest-specific gene (gas6) is a new member of the vitamin K-dependent proteins related to protein S, a negative coregulator in the blood coagulation cascade Mol Cell Biol 1993 13 4976 4985 8336730 10.1128/mcb.13.8.4976-4985.1993 PMC360142

[B3] Hafizi S Dahlbäck B Gas6 and protein S. Vitamin K-dependent ligands for the Axl receptor tyrosine kinase subfamily Febs J 2006 273 5231 5244 10.1111/j.1742-4658.2006.05529.x 17064312

[B4] Stenhoff J Dahlbäck B Hafizi S Vitamin K-dependent Gas6 activates ERK kinase and stimulates growth of cardiac fibroblasts Biochem Biophys Res Commun 2004 319 871 878 10.1016/j.bbrc.2004.05.070 15184064

[B5] Hall MO Obin MS Heeb MJ Burgess BL Abrams TA Both protein S and Gas6 stimulate outer segment phagocytosis by cultured rat retinal pigment epithelial cells Exp Eye Res 2005 81 581 591 10.1016/j.exer.2005.03.017 15949798

[B6] Ishimoto Y Ohashi K Mizuno K Nakano T Promotion of the uptake of PS liposomes and apoptotic cells by a product of growth arrest-specific gene, gas6 J Biochem 2000 127 411 417 10731712 10.1093/oxfordjournals.jbchem.a022622

[B7] Rothlin CV Ghosh S Zuniga EI Oldstone MB Lemke G TAM receptors are pleiotropic inhibitors of the innate immune response Cell 2007 131 1124 1136 10.1016/j.cell.2007.10.034 18083102

[B8] Lu Q Lemke G Homeostatic regulation of the immune system by receptor tyrosine kinases of the Tyro 3 family Science 2001 293 306 311 10.1126/science.1061663 11452127

[B9] Tjwa M Bellido-Martin L Lin Y Lutgens E Plaisance S Bono F Delesque-Touchard N Herve C Moura R Billiau AD Aparicio C Levi M Daemen M Dewerchin M Lupu F Arnout J Herbert JM Waer M Garcia de Frutos P Dahlbäck B Carmeliet P Hoylaerts MF Moons L Gas6 promotes inflammation by enhancing interactions between endothelial cells, platelets, and leukocytes Blood 2008 111 4096 4105 10.1182/blood-2007-05-089565 18156494

[B10] Budagian V Bulanova E Orinska Z Duitman E Brandt K Ludwig A Hartmann D Lemke G Saftig P Bulfone-Paus S Soluble Axl is generated by ADAM10-dependent cleavage and associates with Gas6 in mouse serum Mol Cell Biol 2005 25 9324 9339 10.1128/MCB.25.21.9324-9339.2005 16227584 PMC1265819

[B11] Ekman C Stenhoff J Dahlbäck B Gas6 is complexed to soluble tyrosine kinase receptor Axl in human blood J Thromb Haemost 2010 8 838 844 10.1111/j.1538-7836.2010.03752.x 20088931

[B12] Cohen J The immunopathogenesis of sepsis Nature 2002 420 885 891 10.1038/nature01326 12490963

[B13] Bone RC Balk RA Cerra FB Dellinger RP Fein AM Knaus WA Schein RM Sibbald WJ Definitions for sepsis and organ failure and guidelines for the use of innovative therapies in sepsis. The ACCP/SCCM Consensus Conference Committee. American College of Chest Physicians/Society of Critical Care Medicine Chest 1992 101 1644 1655 10.1378/chest.101.6.1644 1303622

[B14] Borgel D Clauser S Bornstain C Bieche I Bissery A Remones V Fagon JY Aiach M Diehl JL Elevated growth-arrest-specific protein 6 plasma levels in patients with severe sepsis Crit Care Med 2006 34 219 222 10.1097/01.CCM.0000195014.56254.8A 16374177

[B15] Gibot S Massin F Cravoisy A Dupays R Barraud D Nace L Bollaert PE Growth arrest-specific protein 6 plasma concentrations during septic shock Crit Care 2007 11 R8 10.1186/cc5158 17241453 PMC2151874

[B16] Linder A Christensson B Herwald H Bjorck L Akesson P Heparin-binding protein: an early marker of circulatory failure in sepsis Clin Infect Dis 2009 49 1044 1050 10.1086/605563 19725785

[B17] Balogh I Hafizi S Stenhoff J Hansson K Dahlbäck B Analysis of Gas6 in human platelets and plasma Arterioscler Thromb Vasc Biol 2005 25 1280 1286 10.1161/01.ATV.0000163845.07146.48 15790929

[B18] Bellomo R Ronco C Kellum JA Mehta RL Palevsky P Acute renal failure - definition, outcome measures, animal models, fluid therapy and information technology needs: the Second International Consensus Conference of the Acute Dialysis Quality Initiative (ADQI) Group Crit Care 2004 8 R204 212 10.1186/cc2872 15312219 PMC522841

[B19] Sather S Kenyon KD Lefkowitz JB Liang X Varnum BC Henson PM Graham DK A soluble form of the Mer receptor tyrosine kinase inhibits macrophage clearance of apoptotic cells and platelet aggregation Blood 2007 109 1026 1033 10.1182/blood-2006-05-021634 17047157 PMC1785151

[B20] Caraux A Lu Q Fernandez N Riou S Di Santo JP Raulet DH Lemke G Roth C Natural killer cell differentiation driven by Tyro3 receptor tyrosine kinases Nat Immunol 2006 7 747 754 10.1038/ni1353 16751775

